# Preliminary Results of a Combined Score Based on sIL2-Rα and TIM-3 Levels Assayed Early After Hematopoietic Transplantation

**DOI:** 10.3389/fimmu.2019.03158

**Published:** 2020-02-07

**Authors:** Salvatore Leotta, Giuseppe Sapienza, Maria Grazia Camuglia, Giuseppe Avola, Annalia Di Marco, Gaetano Moschetti, Angelo Curto Pelle, Uros Markovic, Giulio Antonio Milone, Alessandra Cupri, Oriana Bianco, Viviana Frontini, Andre Spadaro, Anna Elisa Marchese, Roberto Crocchiolo, Giuseppe Milone

**Affiliations:** ^1^Unità di Trapianto di Midollo, Divisione di Ematologia, Azienda Ospedaliera Policlinico Vittorio Emanuele, Catania, Italy; ^2^Unità Operativa di Oncoematologia e BMT Unit, Istituto Oncologico del Mediterraneo, Catania, Italy; ^3^First Department of Laboratory Medicine, Azienda Ospedaliera Policlinico Vittorio Emanuele, Catania, Italy; ^4^Servizio di Immunoematologia, Ospedale Niguarda, Milan, Italy

**Keywords:** graft-vs.-host disease, Tim 3, sIL2-Rα, cytokines, hematopoietic stem cell transplantation

## Abstract

Assays of cytokines in the plasma at the onset of graft-vs. -host disease (GVHD) can predict disease severity and treatment-related mortality (TRM); however, the optimal time during which cytokines should be tested and the specific panel of cytokines with the highest predictive ability remain unknown. We chose a predefined time point, 18 days after hematopoietic stem cell transplantation (HSCT), to measure the levels of six cytokines in the plasma: soluble interleukin-2 receptor alpha (sIL2-Rα), T-cell immunoglobulin domain and mucin domain-3 (TIM-3), suppression of tumorigenicity-2 (ST-2), intercellular adhesion molecule (ICAM-1), interferon-gamma (IFN-γ), and interleukin-6 (IL-6). The study included 95 patients, who underwent allogeneic hematopoietic transplantation at our institution. Plasma levels of sIL2-Rα and TIM-3, measured as continuous data, had predictive value for overall survival (sIL2-Rα, *p* = 0.002; TIM-3, *p* = 0.0007), while TRM could be predicted by sIL2-Rα (*p* = 0.0005), IFN-gamma (*p* = 0.01), and IL-6 (*p* = 0.0001). No cytokine was associated with the risk of relapse. Patients were categorized into groups, according to cytokine thresholds determined by receiver operating characteristic curve analysis (sIL2-Rα ≤ or > 8,100 pg/ml; TIM-3 ≤ or > 950 pg/ml) and multivariate analysis was conducted. High levels of both TIM-3 and sIL2-Rα were significant predictors of poor survival [TIM-3 > 950 pg/ml: hazard ratio (HR) = 6.214 (95% CI 1.939–19.910), *p* = 0.002 and sIL2-Rα > 8.100 pg/ml: HR = 2.644 (95% CI 1.308–5.347), *p* = 0.006]. Using these cutoff thresholds, we constructed a composite scoring system that could distinguish three different groups of patients with varying rates of TRM: high risk, 41.7%; intermediate risk, 10.8%; and low risk, 7.1% (Gray's test: *p* = 0.001). If confirmed in a validation cohort, this composite scoring system could be used to guide the modulation of post-transplant immune suppressive therapy.

## Introduction

Hematopoietic stem cell transplantation (HSCT) is an effective treatment for patients with hematological tumors; however, its use is limited by the high risk of treatment-related mortality (TRM), which ranges from 15 to 25% (1). The elevated risk of TRM is attributable to the alloreactivity of donor T cells, which contributes to the development of numerous transplant-related complications. The most evident clinical expression of alloreactivity after HSCT is acute-graft-vs.-host disease (a-GVHD). Severe a-GVHD or cortico-refractory a-GVHD is associated with high rates of TRM (2). Patients at high risk of developing cortico-refractory a-GVHD can be identified by assessing cytokine levels in the plasma at the onset of a-GvHD (3).

Biomarker assays with a high predictive value at the onset of a-GVHD include single cytokines, such as soluble suppression of tumorigenicity-2 (sST-2), a protein encoded by the *IL1RL1* gene; interleukin 6 (IL-6); soluble interleukin-2 receptor (sIL-2R); and soluble tumor necrosis factor receptor 1 (sTNFR1) (4–6). Alternatively, a panel of various cytokines can be constructed. An array consisting of sIL2-Rα, sTNFR1, interleukin 8 (IL8), and hepatocyte growth factor (HGF) was proposed by Paczesny et al. (7), while Levine et al. developed an array comprising sTNFR1, sIL2-Rα, and regenerating islet derived protein 3-alpha (REG-3-α) (8). High values of sST-2 and soluble T-cell immunoglobulin domain and mucin domain-3 (sTIM-3) are correlated with both TRM and overall survival (OS) (9).

Moreover, Major-Monfried et al. showed that the Hartwell algorithm, based on serum levels of REG-3 α and s-ST2, when assayed 7 days after the onset of a-GVHD, can stratify patients at risk of 1 year TRM better than other clinical scores (10). The optimal time at which to conduct a predictive cytokine assay may not be at the onset of a-GVHD, and both sTIM-3 and sST-2 have high predictive value for TRM and severe a-GVHD when assayed earlier, on day +7 after transplantation (6, 11).

We hypothesized that a panel of cytokines analyzed on day +18, before the onset of a-GVHD, may be clinically useful in terms of its ability to predict outcome. We chose this time point based on the observation of the presence of biological expression of alloreactivity at that time in patients who later develop clinically overt a-GVHD (12).

## Methods

### Study Design

In this prospective study, we measured plasma levels of sIL2-Rα, TIM-3, ST-2, intercellular adhesion molecule (sICAM-1), IFN-γ, and IL-6 at a fixed time point after allogeneic hematopoietic transplantation; that is, day +18 after allogeneic hematopoietic transplantation.

### Patients

The present study included 95 patients, who underwent allogeneic hematopoietic transplantation at our institution between January 2013 and September 2017. It was a biological study aimed to assess at day +18 an array of cytokines in the plasma, as well as the frequency of clonogenic precursors in marrow aspirates. The study was approved by the Ethical Committee of our institution (35/2013VE), as an observational study. All patients received relevant information and gave consent.

Diagnoses included acute leukemia (*n* = 60), multiple myeloma or lymphoma (*n* = 16), and others (*n* = 19). Diagnoses were grouped into two categories: acute leukemia, lymphoma, and multiple myeloma (AL/LYM/MM) and aplastic anemia, myelodysplastic syndromes, and chronic myeloproliferative neoplasms (AA/MDS/MPN). Conditioning schedules were classified as myeloablative (MA) or reduced intensity conditioning (RIC), according to recently proposed criteria (13). MA conditioning was used in 82.5% of cases, and RIC was used in 17.5%. Intravenous busulfan (12.8 mg/kg), plus either fludarabine 160 mg/m^2^ or cyclophosphamide 120 mg/kg, comprised the most commonly used MA conditioning regimen (42% of all MA conditioning). A further 8% of MA conditioning comprised total body irradiation (12 Gy) plus cyclophosphamide.

In 84% of cases, GVHD prophylaxis was cyclosporine + short course methotrexate (MTX). The MTX was routinely administered in four doses after matched unrelated donor (MUD) transplantation, or transplantation from an identical family donor, from whom the source was hematopoietic progenitor cells obtained from peripheral blood stem cells. Three doses of MTX were administered to patients with transplants from identical family donors, from whom the source was bone marrow. Anti-thymocyte globulin was routinely used only after MUD transplantation. The GVHD prophylaxis was grouped into two categories, CSA + MTX + ATG vs. others forms of prophylaxis. Criteria for acute GVHD scoring and treatment have been previously reported (12). Clonogenic precursors (colony forming unit–granulocyte, monocyte [CFU-GM] and burst-forming unit–erythroid [BFU-e]) in the marrow were studied on day +30 (*n* = 39). Demographic and disease-related features of patients are reported in Table 1. At the time of analysis, median follow-up for patients still alive was 198 weeks (range, 99–344 weeks).

**Table 1:** Patient demographics and transplant-related features.

***n***	**95**
Male	51 (53%)
female	44 (46%)
Age, years (median)	46.0 years (IQR 15.7)
acute leukemia	60 (63%)
myeloma	8 (8.4%)
lymphoma	8 (8.4%)
other diagnosis	19 (20.2%)
HTC-comorbidity score	
0–2	85 (89%)
3–5	10 (10.5%)
early phase	34 (35.5%)
advanced phase	61 (64.5%)
Full myeloablative	78 (82.5%)
Reduced intensity	17 (17.5%)
HLA-identical sibling	39 (41.0%)
MUD	47 (49.5%)
Haploidentical	9 (9.5%)
Source	
BM	51 (53%)
PBSC	44 (46%)
GVHD Prophylaxis	
CSA + MTX + ATG	46 (48%)
CSA + MTX	35 (36%)
CTX post	12 (12.6%)
CSA + 6MP	2 (2%)
BM: Infused CD34+ x10e6/kg	2.6 IQR 2.0
PBSC: infused CD34+ x10e6/kg	6.0 IQR 4.5
BM: *N* engraftment days	20.0 IQR 4
PBSC: *N* engraftment days	17.5 IQR 3.7
Acute GVHD grade 0–1	52 (54.7%)
Acute GVHD grade 2–4	43 (45.2%)

### Cytokine Assay

Blood was drawn on day +18/+19 after transplantation, and plasma was obtained by centrifugation within 2 h. Samples were stored at −70°C until further analysis. Cytokines were assayed by automated ELISA, and each sample was tested in duplicate. A titration curve was constructed for known concentrations of various cytokines in the plasma, obtained from the kit manufacturer. Both ST-2 and TIM-3 were assayed using Bio-Rad ELISA kits, while high sensitivity IFN-γ, IL-6, and sIL2-Rα assays were conducted using Diaclone ELISA kits. The ELISAs were conducted by one of the authors (VF) in a central laboratory at our hospital, which specializes in this type of assay, and data analysis was supervised by AEM. Owing to missing data, TIM-3 and sIL2-Rα results were available for only 75/95 patients.

### Statistical Analysis

Comparisons of cytokine concentrations between groups, or other data with a non-normal distribution, were performed using the non-parametric Mann–Whitney *U*-test. Median and interquartile ranges (IQR) were used to describe the data. The values of cytokines as continuous variables were tested using a Cox proportional hazard model for OS, and a Fine and Gray proportional hazard model for competing events test for TRM and relapse risk (RR).

Receiver operating characteristic (ROC) curves were used to identify cutoff values for cytokine levels and determine the best combination of sensitivity and specificity with respect to OS. These cutoff values were used to divide patients into two groups. Gray's test was used for comparison of the cumulative incidence of competing risks (TRM and RR). A value of *p* ≤ 0.05 was considered to indicate statistically significant differences. Statistical analyses were performed using the StatView 5.0 (Cary, NC) or R software (EZR, version 3.1.1; 2014, R Foundation for Statistical Computing, Vienna, Austria).

## Results

### High Cytokine Levels at Day +18 Are Associated With Low OS and High TRM, but Not With High RR

Plasma levels of cytokines on day +18 are reported in Table 2. Patients with transplants from MUD or haploidentical donors had higher plasma levels of sIL2-Rα (*p* = 0.002), TIM-3 (*p* = 0.009), ICAM-1 (*p* = 0.03), and IL-6 (*p* = 0.05) than those who received transplants from HLA-identical siblings.

**Table 2:** Day +18 cytokines in plasma according to donor type and to HSC source.

	**All patients**	**MUD/HAPLO**	**SIBLINGS**	**BM**	**PBSC**
REC IL2 (pg/ml) Median (IQR)	6,779 (5,980)	7,700 (5,531)	5,100 (4,719)	6,790 (6,633)	6,613 (4,818)
		*P* = 0.002		*P* = 0.47	
TIM-3 (pg/ml) Median (IQR)	1,450 (927)	1,559 (745)	1,172 (924)	1,357 (894)	1,550 (1092)
		*P* = 0.009		*P* = 0.73	
IL6 (pg/ml) Median (IQR)	3.7 (6.2)	4.4 (9.0)	3.4 (2.1)	3.8 (5.5)	3.6 (7.1)
		*P* = 0.05		*P* = 0.85	
IFN-gamma (pg/ml) Median (IQR)	6.2 (12.4)	6.3 (12.6)	5.9 (12.2)	11.7 (12.9)	5.7 (9.2)
		*P* = 0.48		*P* = 0.17	
ST2 (pg/ml) Median (IQR)	22,336 (23,324)	29,800 (21,713)	16,200 (23,480)	22,800 (23,170)	22,256 (23,892)
		*P* = 0.11		*P* = 0.7	
sICAM-1 (ng/ml)	133.5 (129)	168 (145)	107 (119)	126 (105)	170 (163)
		*P* = 0.03		*P* = 0.67	

For all patients under investigation, the OS at 2 years was 58.6% (95% confidence interval [CI]), 48–67%), while the TRM at 2 years was 17.9% (95% CI, 10.9–26.3%). The overall RR at 2 years was 24.2% (95% CI, 16.1–33.3%). Factors important for OS were age (HR = 1.034, *p* = 0.01), marrow as the source of HSCs (HR = 2.053; 95% CI, 1.142–3.691; *p* = 0.01), use of a-GVHD prophylaxis other than CSA + MTX + ATG (HR 1.794; 95% CI, 1.011–3.184; *p* = 0.04) and AA/MDS/MPN diagnosis type (HR = 0.223; 95% CI, 0.054–0.920; *p* = 0.03). In contrast, no significant association was observed between OS and Haplo-MUD donor type, hematopoietic cell transplantation HCT-comorbidity score, disease stage, or conditioning type. When evaluated as continuous data, sIL2-Rα (HR = 1.005, *p* = 0.002) and TIM-3 (HR = 1.054, *p* = 0.0007) were also significantly associated with OS (Table 3). The levels of sIL2-Rα, IFN-gamma, and IL-6 were found to be important predictors of TRM (Table 3). No biomarkers were significantly associated with RR.

**Table 3:** Importance of cytokine levels studied as continuous data for OS, TRM, and relapse rate (univariate analysis).

	**OS**		**TRM**		**RR**	
	**HR** **95% CI**	***P***	**HR** **95% CI**	***P***	**HR** **95% CI**	***P***
Rec IL-2	1.005	0.002	1.046	0.0005	1.000	0.88
	1.002–1.008		1.015–1.078		0.999–1.000	
TIM-3	1.054	0.0007	1.000	0.22	1.000	0.14
	1.027–1.082		0.999–1.001		0.999–1.001	
sICAM-1	0.999	0.16	1.003	0.15	1.002	0.72
	0.999–1.005		0.992–1.006		0.997–1.005	
ST-2	1.000	0.15	1.000	0.91	1.000	0.23
	1.000–1.000		1.000–1.000		1.000–1.000	
IFN-gamma	1.024	0.15	1.060	0.01	0.984	0.48
	0.991–1.057		1.012–1.110		0.919–1.029	
IL-6	1.015	0.05	1.039	0.0001	0.987	0.35
	1.000–1.031		1.023–1.055		0.942–1.014	

### ROC Curves and Identification of Cutoff Levels for Selection of the Most Informative Cytokines for the Prediction of OS

We wished to identify threshold levels of cytokines with clinical importance for predicting OS. Thus, the area under the curve (AUC) was calculated for the ROC curves (Supplementary Table 1) and the best cutoff values were identified. The ROC curve for TIM-3, with regard to the end point of OS, had an AUC of 0.616 (95% CI 0.488–0.744), with a cutoff of 950 pg/ml. The ROC curve for sIL2-Rα had an AUC of 0.605 (95% CI 485–0.726), with a cutoff of 8,100 pg/ml. The ROC curve for IL-6 had an AUC of 0.563 (9% CI 0.434–0.692), with a cutoff of 3,490 pg/ml. The ROC curve for IFN-γ had an AUC of 0.602 (95% CI 0.474–0.730), with a cutoff of 6,360 pg/ml (Supplementary Figures 1, 2).

The predictive power of cytokines for OS was then evaluated by grouping patients, based on these cutoff values. Data were then analyzed using a multivariable stepwise Cox proportional model, which included the variables age, source of HSCs, diagnoses categorized into two groups (AA/MDS/MPN vs. AL/LYM/MM), GVHD prophylaxis, and donor type. Factors significantly associated with OS were HSC source, diagnosis, GVHD prophylaxis, and levels of both TIM-3 and sIL2-Rα above their respective cutoff values (HSC source: *p* to remove, *p* = 0.003; diagnosis: *p* to remove, *p* = 0.01; GVHD prophylaxis other than CSA + MTX + ATG: *p* to remove, *p* = 0.002; TIM-3 over the threshold: *p* to remove, *p* = 0.001; sIL2-Rα over the threshold: *p* to remove, *p* = 0.008).

Patients with TIM-3 and sIL2-Rα levels over these thresholds had lower OS rates, according to univariable and multivariable analyses (Table 4).

**Table 4:** Univariate and multivariable analysis for OS incorporating values of Tim-3 and sIL2-Rα dichotomized in two groups.

	**OS Univariate**			OS Multivariate^***^		
	**HR**	**95% CI**	***P***	**HR**	**95% CI**	***P***
Tim3 over 950 pg/ml	4.699	1.652–13.080	0.003	6.214	1.939–19.910	0.002
REC IL-2 over 8,100 pg/ml	2.762	1.530–4.988	0.0007	2.644	1.308–5.347	0.006
Age	1.034	1.007–1.062	0.01	1.001	0.974–1.028	0.95
Source: Marrow vs. PBSC	2.053	1.142–3.691	0.01	2.328	1.082–5.007	0.03
Diagnosis: MDS/AA/MPN	0.223	0.054–0.920	0.03	0.097	0.013–0.726	0.02
Donor type: MUD-HAPLO vs. HLA-ID SIBLING	1.450	0.814–2.585	0.20	1.100	0.484–2.502	0.82
GVHD prophylaxis: other than CSA + MTX + ATG vs. CSA + MTX + ATG	1.794	1.011–3.184	0.04	2.313	1.030–5.192	0.04
Conditioning regimen FMA vs. RIC	1.038	0.503–2.139	0.92			

*** *Studied in the set of 75 patients in which both data on TIM-3 and REC IL-2 were available*.

### TRM in Patients Grouped According to TIM-3 and sIL2-Rα Cutoff Values

When patients were grouped based on TIM-3 levels, according to the determined cutoff value, those with TIM-3 levels <950 pg/ml had a TRM of 5.3% (95% CI, 0.3–22%) vs. 23.7% (95% CI, 13.7–35.2%) in patients with higher plasma levels of this cytokine (*p* = 0.05). Further, TRM in patients with low levels of sIL2-Rα (<8,100 pg/ml) was 10.5% (95% CI, 4.2–20.1%) vs. 34.5% (95% CI, 17.7–51.9%) in the group with higher plasma levels of the same cytokine (*p* = 0.002).

### Composite Scoring System Based on TIM-3 and sIL2-Rα Levels

Given the importance of sIL2-Rα and TIM-3 for OS and TRM, we constructed a composite scoring system, based on the frequency with which the two biomarkers showed levels over their respective threshold values. A score of 0 was attributed to patients with both TIM-3 and sIL2-Rα levels below the respective cutoff values; a score of 1 was attributed to patients with levels of only one of the two biomarkers over the threshold; and a score of 2 was attributed to patients with levels of both biomarkers above the respective cutoff values.

**Figure 1:**
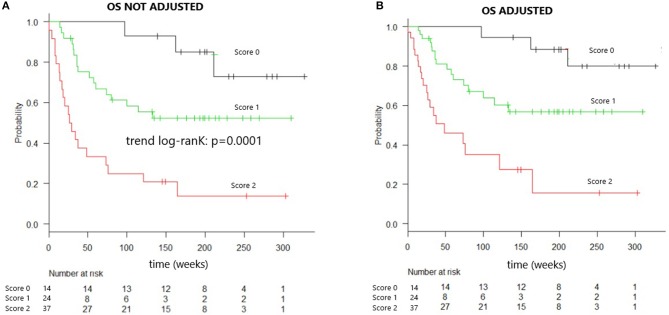
Overall survival of patients identified by combined cytokines score. **(A)** Overall survival curve of patients identified by combined cytokines score. Overall survival at 2 years is 95% for patients “score 0,” 65% for “score 1” patients, 30% for “score 2” patients (trend log-rank: *p* = 0.0001), median OS in score 2 patients is 26 weeks. **(B)** Overall survival curve of patients identified by combined cytokines score and adjusted for the effect of diagnosis, GVHD prophylaxis, and HSC source.

Kaplan–Meier analysis evaluating OS in patients grouped according to the composite score, both unadjusted and adjusted, for the effects of diagnosis, GVHD prophylaxis, and HSC source, is presented in Figure 1. The OS rates were projected to be 95, 65, and 30% at 2 years in patients with scores of 0, 1, and 2, respectively (trend log-rank, *p* = 0.0001). Multivariate analysis indicated that patients with both sIL2-Rα and TIM-3 levels over the respective threshold values had an HR of 4.188 (95% CI, 1.948–9.004) for death, relative to all other patients (*p* = 0.0002) (Table 5).

**Table 5:** Evaluation of importance of the composite score for OS, using Cox proportional hazard multivariate analysis.

	**Univariate for OS**			Multivariate for OS^***^		
	**HR**	**95% CI**	***P***	**HR**	**95% CI**	***P***
Both IL-2 rec and TIM-3 over the cutoff vs. all other patients	4.089	2.179−7.673	0.0001	4.188	1.948–9.004	0.0002
Age (continuous data)	1.034	1.007–1.062	0.01	1.012	0.986–1.039	0.37
Source (Marrow vs. PBSC)	2.053	1.142–3.691	0.01	1.860	0.852–4.061	0.11
Donor type (MUD-HAPLO vs. HLA-ID SIBLING)	1.450	0.814–2.585	0.20	1.300	0.567–2.982	0.53
Diagnosis MDS/MPN/AA vs. Others	0.223	0.054–0.920	0.03	0.131	0.018–0.971	0.04
GVHD prophylaxis other than CSA+MTX+ATG	1.794	1.011–3.184	0.04	2.551	1.138–5.717	0.02

*** *Studied in the set of 75 patients in which both data on TIM-3 and REC IL-2 were available*.

**Figure 2:**
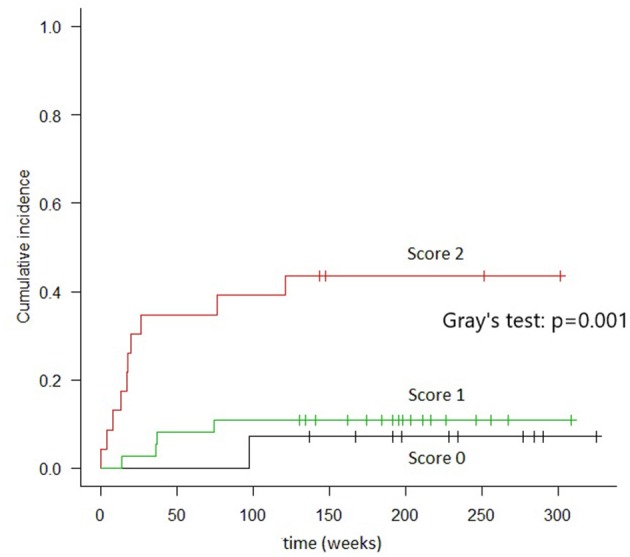
Cumulative incidence of TRM in groups of patients identified by combined cytokines score. In “score 0” patients (both TIM-3 AND sIL2-Rα below the threshold) the TRM, at 2 years, resulted 7.1%. In “score 1” patients (either TIM-3 or sIL2-Rα over the threshold), the TRM was 10.8%. In “score 2” patients (both TIM-3 and sIL2-Rα over the threshold), TRM was 41.7% (Gray's test: *p* = 0.001).

Patients with scores of 0 (*n* = 14) had 2 years TRM rates of 7.1% (95% CI, 4–28%); those with scores of 1 (*n* = 37) had 2 years TRM rates of 10.8% (95% CI, 3.4–23.3%); and those with scores of 2 (*n* = 24) had 2 years TRM rates of 41.7% (95% CI, 21.5–60.7%) (Gray's test, *p* = 0.001) (Figure 2). Fulfillment of criteria for score 2 had a sensitivity of 0.688 (95% CI, 0.413–0.890) and a specificity of 0.780 (95% CI, 0.653–0.877) in predicting TRM (Table 6).

**Table 6:** Accuracy of the prediction of TRM by identification of score 2 patients (criteria “TIM3 over 950 ng/ml and sIL-2rec over 8,100 ng/ml” assayed at Day +18).

	**TRM yes**	**TRM no**	**Totel**
Criteria for SCORE 2 fulfilled	11	13	24
Criteria for SCORE 2 not fulfilled	5	46	51
Total	16	59	75
**Point estimates and 95% CIs:**			
	**Estimation**	**Lower CI**	**Upper CI**
Apparent prevalence	0.320	0.217	0.438
True prevalence	0.213	0.127	0.323
Sensitivity	0.688	0.413	0.890
Specificity	0.780	0.653	0.877
Positive predictive value	0.458	0.256	0.672
Negative predictive value	0.902	0.786	0.967
Diagnostic accuracy	0.760	0.647	0.851
Likelihood ratio of positive test	3.120	1.742	5.588
Likelihood ratio of negative test	0.401	0.191	0.840

**Figure 3:**
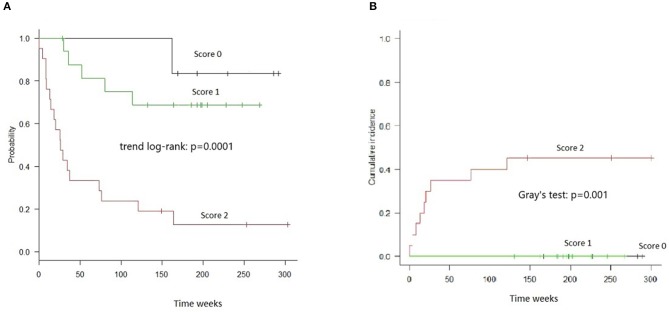
OS and TRM in HAPLO-MUD transplants in groups of patients identified by combined cytokines score. **(A)** OS resulted significantly different (trend log-rank: *p* = 0.0001); score 2 patients had a median survival of 26 weeks. **(B)** TRM evaluated by Gray's test resulted significantly different (*p* = 0.001).

The combined score was also highly informative in the stratum of Haplo-MUD transplantation, both with respect to OS (Trend log-rank: *p* = 0.0001) and TRM (Gray's test *p* = 0.001) (Figure 3).

Results were not different if analysis was performed in the stratum of patients affected by acute leukemia. In fact, when we selected AML, ALL, and MDS patients (n 65), group 0 patients had, at 2 years, an OS, of 92.9% (CI 59.1–99.0%) and a TRM of 7.1% (CI 0.4–28.5%); group 1 patients had an OS of 58.3% (CI 40.6–72.4%) and a TRM of 10.8% (CI 3.4–23.3%); group 2 patients had an OS of 20.8% (CI 7.5–38.5%) and a TRM of 45.8% (CI 24.7–64.7%). Difference in OS was significant (log-rank: *p* = 0.00004) as difference in TRM was significant (Gray's test *p* = 0.001).

The ROC curve of this combined score had an AUC of 0.738 (95% CI, 0.637–0.840) for OS and an AUC of 0.744 (95% CI, 0.612–0.875) for TRM (Supplementary Figure 3).

### Composite Score and Clinical Features at Day +18

The incidence of Grades II–IV a-GVHD during the first 100 days was higher in patients with scores of 2 vs all other patients (54 vs 36%, respectively); however, this difference was not significant. Further, the incidence of lower gastrointestinal tract involvement in GVHD was 33, 8.5, and 7.1% in patients with scores of 2, 1, and 0, respectively (*p* = 0.007) (Supplementary Table 2).

Patients presenting with scores of 2 at day +18 had a reduced number of total lymphocytes relative to all other patients (0.050 × vs. 0.220 ×10^9^/L, respectively; *p* = 0.0001), as well as a lower median absolute neutrophil count (0.345 × vs. 0.540 ×10^9^/L, *p* = 0.04). Evaluation of BFU-e growth, using marrow samples collected on day +30 in 39 patients, revealed significantly reduced levels of BFU-e in patients with scores of 2 (*p* = 0.005) (Supplementary Table 2). Score 2 patients had, in respect to all other patients, a higher need for blood red cell transfusion, median 5 units vs. 2 units (*p* = 0.009). A higher number of platelets transfusion were registered in score 2 patients, median 9 units vs. 4 units (*p* = 0.0002). Moreover, patients with scores of 2 had higher rates of fever between days +10 and +18 (58, vs. 45 and 14% in patients with scores of 1 and 0, respectively; *p* = 0.02). No differences were detected in the incidence of positive hemoculture (*p* = 0.70) or cytomegalovirus (CMV) reactivation rate during the first 25 days (*p* = 0.52) among the three groups.

Among the patients with scores of 0–1 (*n* = 51 patients), 20 died and five of those deaths were due to TRM. Of those five deaths due to TRM, four were ascribed to a-GVHD or infections. Among patients with scores of 2 (*n* = 24), 20 died, and 11 of those deaths were due to TRM; of those 11 deaths, eight were ascribed to a-GVHD or infections. Thus, deaths due to a-GVHD or infections were recorded for 4/51 (8%) patients in the group with scores of 0–1, and 8/24 (33%) in the group with a score of 2 (chi-squared, *p* = 0.001).

## Discussion

In the present study, we found that high plasma levels of the two biomarkers, sIL2-Rα and TIM-3, at a predefined time point (+ 18 days after transplantation) were predictive of increased TRM and low OS. Using cutoff levels of these biomarkers, determined by ROC analysis (TIM-3 > 950 pg/ml and sIL2-Rα > 8,100 pg/ml), we were able to distinguish three separate groups: a high-risk group (patients with levels of both biomarkers above threshold levels), an intermediate-risk group (patients with only one biomarker above its threshold level), and a low-risk group (patients with both biomarkers below the cutoff levels). The TRM rates in the three groups were 41.7, 10.8, and 7.1% in the high-, intermediate-, and low-risk groups, respectively. The difference in mortality between the high-risk and low-risk groups, according to our scoring system, was notable. Our findings suggest that this system could be useful for guiding both pre-emptive and intensified first-line treatment in high-risk patients who develop GVHD and modulating immunosuppression by rapid de-escalation in low-risk patients (14). A discrete heterogeneity was present in our series of patients; however, the importance of our combined score on OS was maintained also when we analyzed subgroups homogeneous in diagnosis (only acute leukemia patients), in donor type (excluding transplants from a haploidentical donor), or in concomitant immunosuppressive treatment (excluding patients already in corticosteroid when blood was drawn for cytokine assay).

Both IL-2 and sIL2-Rα play central roles in the pathogenesis of GVHD. The levels of sIL2-Rα in the early stages of disease show a clear correlation with the incidence of Grades II–IV GVHD and TRM (15–17). In a panel comprising HGF, IL-8, TNFR1, and IL-2Rα, the latter two biomarkers were the most accurate predictors of a-GVHD occurrence. Moreover, only sIL2-Rα predicted response to treatment at 4 weeks (7). In a recent study of T-cell depleted allo-HSCT, sIL2-Rα, in combination with four other markers (elafin, REG3-α, sTNFR-1, and HGF), were included in a scoring system that correlated with a-GVHD severity (9); however, despite the high sensitivity of sIL2-Rα as a predictor of GVHD and TRM, it yielded low specificity. This was likely due to its possible involvement in inflammatory processes other than GVHD, such as veno-occlusive disease, sepsis, and CMV reactivation (18–20). Nevertheless, different results, in this regard, have been obtained by other authors (21). Many complications of HSCT may be related to alloreactivity and share the common pathogenetic denominator of endothelial damage (22, 23). Recent studies have been focused on the prevention of endothelial damage, and consequently its complications, by measuring biomarkers for GVHD (24).

As a cytokine involved in immune regulation, TIM-3 is expressed on activated T cells. Binding of TIM-3 to its ligand results in the inhibition of T-cell proliferation, cytotoxicity, and induction of apoptosis. The soluble form of TIM-3 (sTIM-3) interferes with immune regulation and plays a significant role in the pathogenesis of GVHD (25). Elevated levels of sTIM-3 have been found in plasma samples from patients with GVHD, and is a strong predictor of mid-gut GVHD (25). In a study by McDonald (6), TIM-3 was one of the most informative biomarkers for Grades III–IV a-GVHD and TRM at 1 year.

In the present study, high-risk patients with a score of 2 had a higher frequency of a severe pattern of a-GVHD that included the presence of lower gut involvement. Thus, our results confirm what has already been reported by Hansen et al. (25). We found no association between CMV reactivation or sepsis and a score of 2 (the high-risk group). We also observed that on day +18, patients with a score of 2 had a reduced lymphocyte count. The association between a reduced lymphocyte count and high plasma cytokine levels may partially explain the significance of a poor prognosis with a reduced lymphocyte count (26, 27). Further, in high-risk patients with a score of 2, in addition to the delay in lymphocytic recovery, we observed reduced marrow function. This may suggest that early damage in the marrow microenvironment could be the determining mechanism of both these findings.

One limitation of our scoring system was the lack of validation in an independent set of patients. Such validation will be required before the system can be considered for clinical implementation.

In conclusion, we found that an assay of cytokine levels at day +18 was highly informative. In addition, the combined assessment of TIM-3 and sIL2-Rα levels at that time could be useful for the identification of subgroups with substantial differences in TRM and OS.

## Data Availability Statement

The datasets generated for this study are available on request to the corresponding author.

## Ethics Statement

The studies involving human participants were reviewed and approved by Comitato Etico Policlinico di Catania. The patients/participants provided their written informed consent to participate in this study.

## Author Contributions

GMi designed the study, performed statistical analysis, and wrote the manuscript. SL contributed to writing of the manuscript. RC contributed to the statistical analysis. MC, GMo, and GA collected and stored blood samples. AEM and VF analyzed the cytokine levels using ELISA. GS, ACP, GAM, AC, AS, UM, and OB cared for the patients during transplantation. AM input data to the database.

### Conflict of Interest

The authors declare that the research was conducted in the absence of any commercial or financial relationships that could be construed as a potential conflict of interest.

## Supplementary Material

Supplementary Figure 1 ROC analysis of plasmatic values of s-IL2-R in respect of survival. AUC is 0.605 (CI 0.485–0.726), the 8,100 pg/ml cut-off has sensitivity of 0.46 and a specificity of 0.80.

Supplementary Figure 2 ROC analysis of plasmatic values of Tim-3 in respect of survival. AUC is 0.616 (95% CI: 0.488–0.744), the 950 pg/ml cut-off has sensitivity 0.90 specificity 0.40.

Supplementary Figure 3 ROC analysis of the combined score in respect to TRM. AUC is 0.744 (95% CI: 0.612–0.875).

Supplementary Table 1 AUC of ROC curves of each cytokine in regard of OS and of TRM.

Supplementary Table 2 Clinical and hematological features in patients grouped according to composite score.
