# Supplementary figures and images for: Preliminary Results of a Combined Score Based on sIL2-Rα and TIM-3 Levels Assayed Early After Hematopoietic Transplantation
Source: Front Immunol. 2020 Feb 7;10:3158. doi: 10.3389/fimmu.2019.03158 (PMC7020780; doi:10.3389/fimmu.2019.03158)

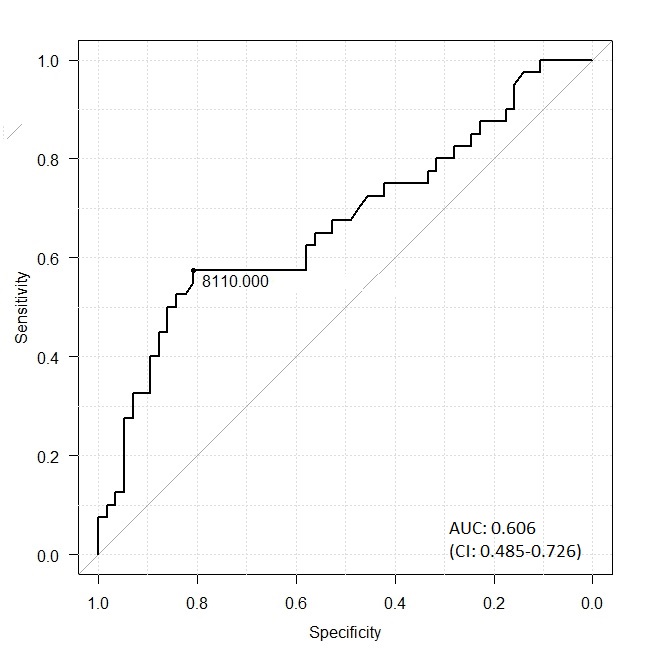

Supplement: Supplementary Figure 1 — ROC analysis of plasmatic values of s-IL2-R in respect of survival. AUC is 0.605 (CI 0.485–0.726), the 8,100 pg/ml cut-off has sensitivity of 0.46 and a specificity of 0.80. [file Image_1.JPEG]

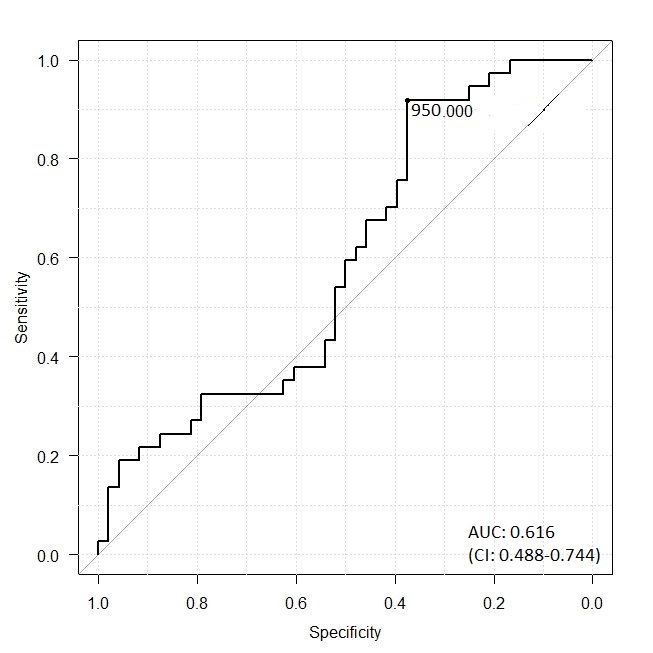

Supplement: Supplementary Figure 2 — ROC analysis of plasmatic values of Tim-3 in respect of survival. AUC is 0.616 (95% CI: 0.488–0.744), the 950 pg/ml cut-off has sensitivity 0.90 specificity 0.40. [file Image_2.JPEG]

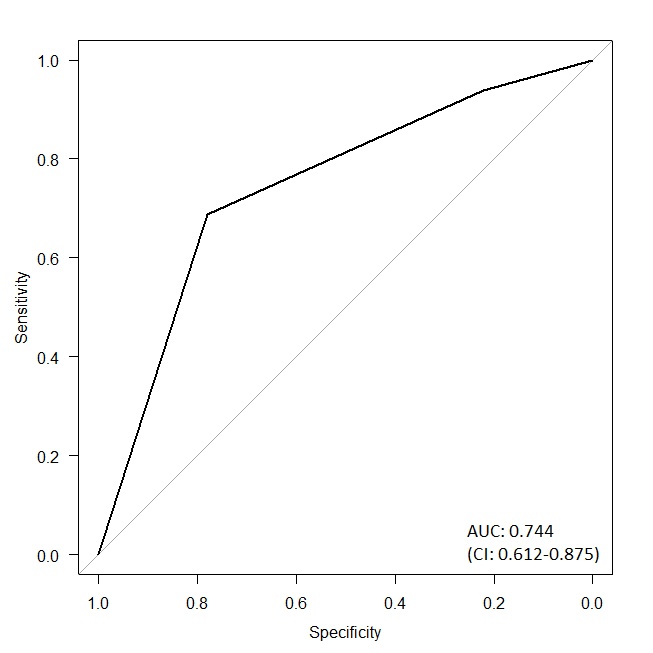

Supplement: Supplementary Figure 3 — ROC analysis of the combined score in respect to TRM. AUC is 0.744 (95% CI: 0.612–0.875). [file Image_3.JPEG]
