# Supplementary material for: Preliminary Results of a Combined Score Based on sIL2-Rα and TIM-3 Levels Assayed Early After Hematopoietic Transplantation
Source: Front Immunol. 2020 Feb 7;10:3158. doi: 10.3389/fimmu.2019.03158 (PMC7020780; doi:10.3389/fimmu.2019.03158)
Supplement: Supplementary Table 1 — AUC of ROC curves of each cytokine in regard of OS and of TRM. [file Table_1.doc]

Supplementary Table 1.

|  | OVERALL SURVIVAL | | TRM | |
| --- | --- | --- | --- | --- |
|  | AUC and 95% CI | | AUC and 95% CI | |
| IL6 | 0.563 | 0.434-0.692 | 0.624 | 0.469-0.779 |
| IFN-gamma | 0.602 | 0.474-0.730 | 0.688 | 0.549-0.826 |
| sIL2R-alpha | 0.605 | 0.485-0.726 | 0.694 | 0.540-0.847 |
| ST-2 | 0.580 | 0.450-0.709 | 0.486 | 0.318-0.653 |
| TIM3 | 0.616 | 0.488-0.744 | 0.572 | 0.431-0.712 |
| sICAM-1 | 0.565 | 0.435-0.695 | 0.621 | 0.472-0.771 |
| SIL2-R-alpha  and TIM3  in a combined  score | 0.738 | 0.636-0.839 | 0.744 | 0.612-0.875 |

AUC of ROC curves of each cytokine in regard of OS and of TRM
