# Supplementary material for: Preliminary Results of a Combined Score Based on sIL2-Rα and TIM-3 Levels Assayed Early After Hematopoietic Transplantation
Source: Front Immunol. 2020 Feb 7;10:3158. doi: 10.3389/fimmu.2019.03158 (PMC7020780; doi:10.3389/fimmu.2019.03158)
Supplement: Supplementary Table 2 — Clinical and hematological features in patients grouped according to composite score. [file Table_2.doc]

Supplementary Table 2

|  | Score 0 Group | Score 1 Group | Score 2 Group |
| --- | --- | --- | --- |
| Serum Total Bilirubin day +18 (mg/dl)  Median (IQR) | 0.735  (0.400) | 0.800  (0.480) | 0.870  (0.740) |
|  | p=0.15* | | |
| PB Lymphocytes  day +18 (x10e9/L)  Median (IQR) | 0.220  (0.210) | 0.270  (0.335) | 0.055  (0.095) |
|  | p=0.0001* | | |
| PB Neutrophil count  Day +18 (x10e9/L)  Median (IQR) | 0.800  (1.890) | 0.485  (960) | 0.345  (565) |
|  | p=0.04* | | |
| PB Neutrophil count  Day +24 (x10e9/L)  Median (IQR) | 1.970  (6.087) | 1.600  (3.210) | 1.095  (1.580) |
|  | p=0.04* | | |
| Frequency of marrow BFU-e at day +30  Median (IQR) | 54./10e5 cells  (16.) | 56./10e5 cells  (41.) | 23./10e5 cells  (14.) |
|  | p=0.005* | | |
| Fever between day +10 and +18 | 14%  (2/14) | 45%  (17/37) | 58%  (14/24) |
|  | Chi-square p=0.02 | | |
| Positive blood culture  between day +10 and +18 | 21%  (3/14) | 33%  (12/37) | 32%  (8/24) |
|  | Chi-square p=0.70 | | |
| CMV antigenemia positive test in PB day +18/+25 | 7.1%  (1/14) | 18.9%  (7/37) | 20.8%  (5/24) |
|  | Chi-square p=0.52 | | |
| a-GVHD Grade II–IV  at start of treatment | 35%  (5/14) | 37%  (14/37) | 54%  (13/24) |
|  | Chi-square p=0.38 | | |
| a-GVHD with lower GI tract =/> Grade I° | 7.1%  (1/14) | 8.5%  (3/35) | 33%  (8/24) |
|  | Chi-square: p=0.007 | | |
| a-GVHD skin rash extension at start of treatment  Mean (IQR) | 23%  (20) | 17%  (16.2) | 20%  (15) |
|  | p=0.48* | | |

Clinical and hematological features in patients grouped according to composite score

*Mann-Withey test comparing score 2 patients with all others (score 0 + score1).

GI: Gastrointestinal
